# Quiz Questions

**Published:** 1885-11

**Authors:** 


QUIZ QUESTIONS.—Course on Dental Patholo y and Therapeutics, Philadelphia Dental College. Prof. J. Foster Flagg, D. D. S. Answered by William C, Foulks, D. D. S., formerly demonstrator and instructor in the Philadelhia Dental College. Third edition. Revised and enlarged. Publishers: The S S. White Dental Manufacturing Co., Philadelphia, New York, Boston and Chicago.

This excellent work is again offered to the dental profession for reference in daily office practice, in a revised and enlarged form which considerably adds to its value. While many, perhaps, may take issue with the "new-departure views" of Prof. Flagg, yet all will agree as to the usefulness of the work in question, and the great assistance it will afford in daily office practice as a work for reference.

Beginning with questions on general principles, it includes the treatment of pathological conditions of the teeth and adjoining structures, and ends with medicaments

